# Help seeking for antibiotics; is the influence of a personal social network relevant?

**DOI:** 10.1186/s12875-019-0955-2

**Published:** 2019-05-14

**Authors:** J. Ellis, I. Vassilev, A. Kennedy, M. Moore, A. Rogers

**Affiliations:** 1 NIHR CLAHRC Wessex, School of Health Sciences, University of Southampton, Building 67, University Road, Southampton, SO17 1BJ UK; 2 NIHR CLAHRC Wessex, Primary Care and Population Science, Aldermoor Health Centre, Aldermoor Close, Southampton, SO16 5ST UK

**Keywords:** Social network, Social support, Primary care, Antibiotic, Antimicrobial

## Abstract

**Background:**

Health policy focuses on reducing antibiotic prescribing that in order to succeed requires the public to hold similar attitudes towards judicious use. Social network influences on health behaviour and attitudes are well established and yet these influences are not sufficiently acknowledged in the UK’s antibiotic stewardship programmes. Therefore, the aim of this study was to evaluate individuals’ attitudes and behaviours towards antibiotics and also identify the social network influences on these in the process of help seeking for self-limiting illnesses.

**Methods:**

From a social network approach the methods used were a personal community mapping exercise which was carried out ahead of a semi-structured interview. A purposive sample was drawn from across the Wessex region and participants were recruited via GP practices and pharmacists. In total 14 adults, and 10 parents of children, who had received a prescription for antibiotics for a self-limiting illness within the 3 months preceding the interview were recruited and interviewed.

**Results:**

Three network types were identified; diverse, family and friend and restricted. The type of network an individual has appears to have an influence on antibiotic attitudes and behaviours. Most notably, the more diverse a network the more likely the individual will delay in help seeking from healthcare professionals as they draw upon self-care strategies advised by network members. The role of the GP varies according to network type too. Individuals’ with diverse networks draw upon GP network members to provide clarity and certainty following a period of self-care. People with restricted networks are more reliant upon the GP, seek help quicker and also more likely to prioritise the GPs advice over other sources of information.

**Conclusion:**

The understanding a social network approach brings to help seeking behaviour for antibiotics could help practitioners modify their consultation approach to mitigate some uncertainties and perceptions around prescribing behaviour.

**Electronic supplementary material:**

The online version of this article (10.1186/s12875-019-0955-2) contains supplementary material, which is available to authorized users.

## Background

Reducing the prescribing rates of antibiotics in primary care [1] is a key policy strategy in the management of the global health risk posed by antimicrobial resistance (AMR). In the UK primary care is both the site of 74% of all antibiotic prescriptions [2], and the focus of targeted management strategies promoting judicious use of antibiotics. Strategies include promoting antibiotic guardians [3], the issuing of delayed prescriptions [4], and TARGET toolkit [5]. Such strategies have had some success, for example by January 2017 42′000 people had pledged to become antibiotic guardians [6]. There is also evidence that the strategy of adopting delayed prescribing is effective in reducing the use of antibiotics. Fewer than 40% of patients issued a delayed prescription go onto use antibiotics [7]. Although others argue a no prescribing policy results in lower uptake [8], patients in receipt of a delayed prescription are also less likely to re-consult [9–11]. While UK policy promotes judicious use of antibiotics and requires healthcare professionals (HCPs) to reduce prescribing rates, it follows logically that the public must hold similar attitudes towards this judicious use if the desired benefits are to be fully achieved. The social network influences on an individual’s attitudes and health behaviours are well established [12], and yet they are not sufficiently acknowledged in the antibiotic stewardship programme.

Take first the decision to consult for antibiotics the influence of social networks members has not been fully considered. The literature indicates that patient expectations of antibiotics are a cited reason for prescribing [13], but little is known about how such apparent expectations are formed. What is known is that where health and wellbeing are concerned help-seeking behaviour is influenced by many factors [14, 15], including context and social relations [16]. We know also know the social network influence of non-healthcare professional members of a network who are instrumental in help seeking, are also triggers for consultation and have an impact on medication taking [17, 18]. Help seeking behaviour is one factor related to antibiotic use, and subsequently connected to this is the possible social influences of network members present in people’s personal communities of support. It is established that through relationships individuals have access to resources, and gain information that may influence their decision to seek help. Furthermore, these relationships can also influence the development and enactment of behaviours and practices [19, 20]. That is, in an antibiotic context tthrough contact with others in a personal network individuals can access knowledge and resources that may challenge, rather than reinforce, their opinion of antibiotics and thus, influence the rationale for using (or not using) a prescription.

Finally, looking at social network influences on antibiotic use is important when the contagion effect is acknowledged. The contagion effect is a significant consideration for campaigns and strategies to tackle AMR that could result in an increased awareness of antibiotic use and of antimicrobial resistance among the general public. We know the effects of contagion in networks are seen in matters of smoking cessation [21], obesity [22] and happiness [23], as well as in phenomena associated with population measures in public health such as MMR vaccine uptake [24]. It is possible that this is relevant in devising an effective population level strategy for antibiotic prescribing for the reverse, that is; the reduction of a practice (i.e. antibiotic use).

In view of what is already established in regard to the role of kinship and other social networks in health matters, it is prudent to consider the role of social networks in relation to the reduction of antibiotic use.

## Method

### Aim

The aim of this study is to provide an evaluation of individuals’ attitudes and behaviours towards antibiotics and the social network influences on these in the process of help seeking for self-limiting illnesses.

### Design

Semi-structured interviews with personal community mapping exercise were carried out. The personal mapping exercise is an established technique [25–27]; it includes a set of concentric circles of importance and encourages discussion and reflection as the exercise is completed [28].

### Participants and recruitment

The study made use of purposive a sampling strategy in order to ensure participants had recently been faced with deciding whether, or not, to take antibiotics. The purposive sample was selected against an inclusion criteria that determined participants had to be either ≥16 years, have good understanding of the English language, and be able to give informed consent. Participants must have also received a prescription for antibiotics in the last 3 months. In addition, parents of individuals aged ≤15 years who had received a prescription for antibiotics in the last 3 months could participate if they too fulfilled the other aspects of the inclusion criteria.

The two recruitment strategies spanned the Wessex region in order to encourage diversity in the sample. The first strategy involved pharmacists (inner city and rural community) who displayed posters and attached a leaflet to the bag of antibiotics being dispensed. The second strategy utilised local GP databases where five GP practices sent an invitation attached to a participant information sheet directly to people who appeared on the GP data base to satisfied the inclusion criteria. Both strategies required the individual to contact the research team to express interest, where at this juncture JE checked the individual fulfilled the inclusion criteria. JE relied on honesty in response to the question, have you or your child received a prescription for antibiotics in the last 3 months. Further details of the study were verbally given to those who satisfied the inclusion criteria and a date for the interview set for those individuals were able and wished to take part.

Recruitment was iterative, and efforts were made to recruit a balance of parents of children who had been prescribed antibiotics and adults who had themselves been prescribed antibiotics. Of the 31 people who contacted the research team to express interest in participation, 24 people took part in the study in total. Despite efforts to recruit in diverse areas, all participants identified themselves as White British, and lived in areas of low deprivation. In total 10 parents and 14 adult patients took part in the study, of these 21-people received a prescription for antibiotics for either a suspected respiratory tract infection (RTI) or urinary tract infection (UTI), and three individuals were on a daily course of antibiotics as part of treatment of a chronic condition. See Table 1 for a summary of participant information.

**Table 1:** Participant information

Participant	Patient or Parent	Age (of patient)	Gender	Antibiotics prescribed for
Bev	Patient	50–59	F	Kidney infection
Florence	Patient	25–29	F	RTI
Emilia	Patient	30–39	F	Unspecified
Dianne	Patient	70–79	F	RTI
Ruth	Parent	15–17	F	Skin infection
Simon	Parent	70–79	M	LTC
Pearl	Patient	70–79	F	UTI
Rachel	Parent	0–4	F	Impetigo
Rose	Patient	80–89	F	UTI
Gail	Parent	0–4	F	RTI
Lynn	Parent	5–9	F	UTI
Emma	Parent	0–4	F	RTI
Victoria	Patient	60–69	F	UTI
John	Patient	40–49	M	LTC
Betty	Patient	85+	F	RTI
Anna	Patient	70–79	F	UTI
Charlotte	Parent	0–4	F	RTI
Sue	Parent	5–9	F	RTI
Caroline	Parent	5–9	F	Eczema
Sophie	Parent	5–9 (×2)	F	RTI
Hugh	Patient	68	M	LTC
Laura	Patient	39	F	UTI
Pete	Patient	75	M	LTC
Ronald	Patient	73	M	RTI

### Data collection

Semi-structured interviews with personal mapping technique took place between September 2016 and March 2017. The mapping technique was used to explore the individual’s personal network and the influence of network members on the use of antibiotics. The exercise was completed directly ahead of the interview in order to encourage participants to consider their personal networks. Participants were asked to map their social network members on the circles in response to the questions “who is most important to you in relation to managing your health when you’re feeling unwell”, and “who is important to you when you make a decision about whether or not to use antibiotics”. The most important network members should be placed close to the ego (centre), and the further away a network member is placed the less important they are (compared to those closer). The interview immediately followed the mapping exercise. The interview guide (available as an Additional file 1) was developed for this study to encourage discussions around the role and importance of network members, the individual’s attitudes and behaviours towards antibiotics drawing on their most recent experiences, as well as the influences of the identified network members. Interviews were conducted by JE either at the participants home or on University premises (as deemed convenient for the participants). All interviews were audio recorded, transcribed verbatim by a transcription service and JE checked for accuracy.

### Data analysis

Data analysis was an iterative process informed by Grounded Theory [29]. Inductive in nature a thematic analysis technique was employed. Moving back and forth between the data, and data collection, the purpose was to move from the specific to the general through identifying and interpreting themes, and then seeking relationships in the data [30]. The first two stages of familiarisation and coding were undertaken by JE [31]. In order to promote rigor JE worked closely with the other authors to complete the subsequent stages of modifying the framework and interpretation. Data collection ceased when data saturation was felt to have been reached; when the framework no longer required modification. Interview transcripts were transported into the software programme NVIVO 10 to assist in data organisation during analysis.

**Fig. 1:**
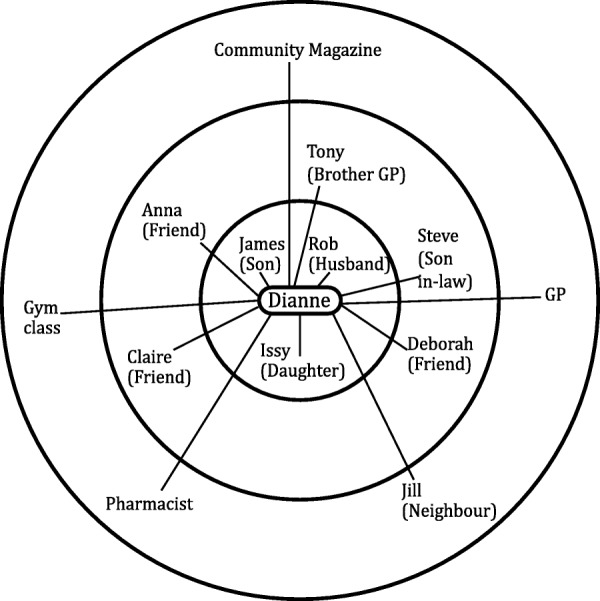
Diverse network

**Fig. 2:**
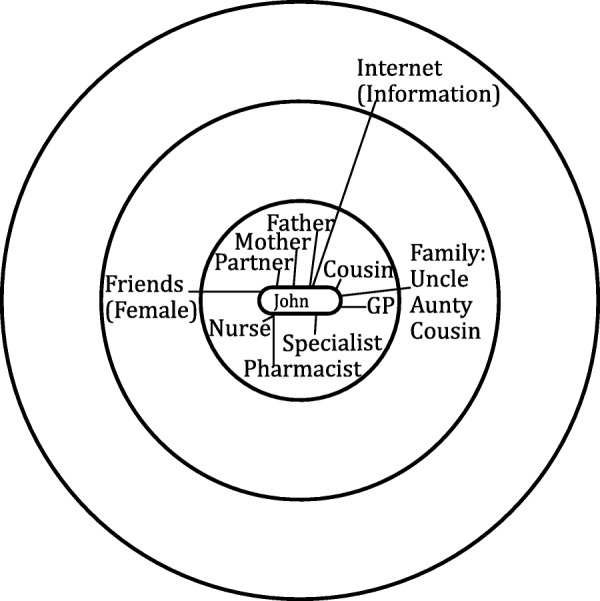
Family and friend centred network

**Fig. 3:**
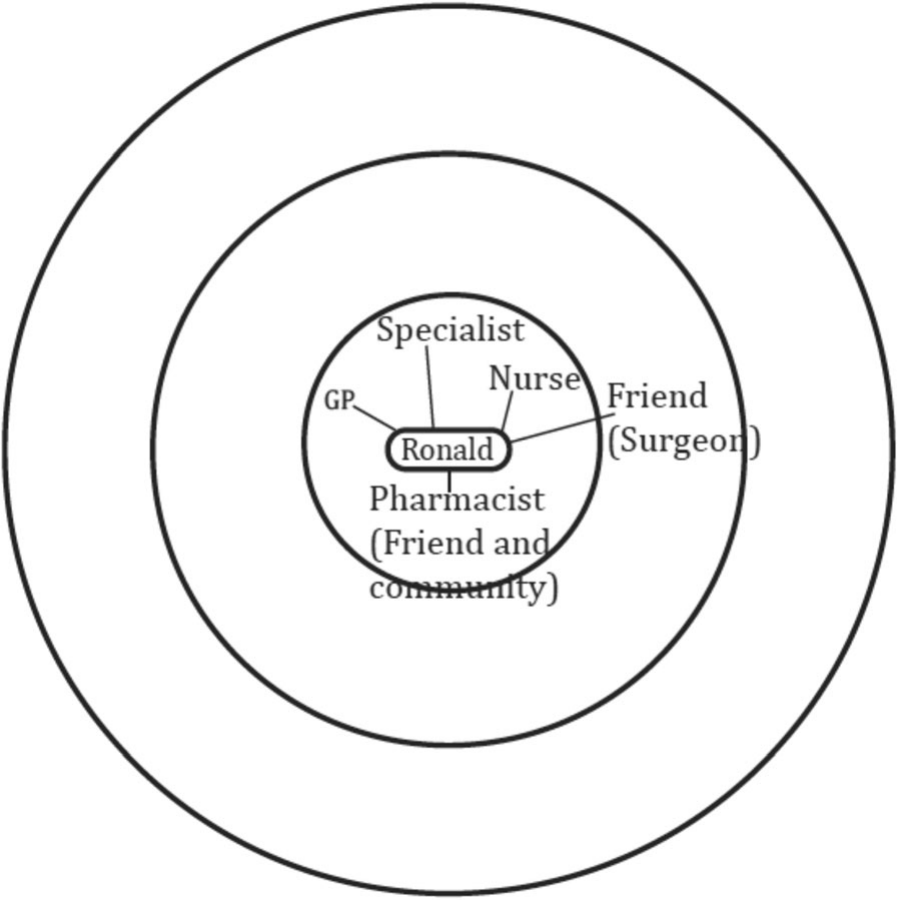
Restricted network

The personal network maps were not imported into NVIVO but were instead interpretively analysed by drawing upon established network typologies as guidance [20, 32, 33]. Previous work of the authors has established key characteristics across network typologies [19, 34]. The most common characteristics are type of relationships and frequency of contact with network members, as well as the size of the network. Based on these characteristics three network types were distinguished. Diverse networks are characterised by higher numbers of relatives and friends with whom regular contact is maintained, and also includes hobby groups (sports teams, choir etc.) or faith groups (any religious affiliation / place of worship) (see Fig. 1). Family and friend centered networks are made up of relatives and friends with whom regular contact is kept, and it is distinguished from diverse networks by an absence of hobbies and groups (see Fig. 2). Restricted networks have low levels of contact with few friends and relatives (see Fig. 3).

## Results

In total, the 24 respondents identified 162 network connections, 49 of whom were relatives, 58 friends (including colleagues) and 39 connections to the healthcare system, in addition to the 16 ‘other’ connections that include the internet and media as information sources. More people had family and friend centred (12) networks than diverse (8) or restricted (4) network types. The type of network, as presented in Table 2, appears to have an influence on antibiotic behaviour and attitudes. Most notably, the more diverse a network the more likely the individual will delay in help seeking as they gain support from family or friends. Furthermore, in general the more diverse a network the fewer formal healthcare professional (HCP) ties are present. The emerging network effect is discussed in the three main themes. The first is ‘rehearsed self-care strategies’ and the second is ‘the temporary and strategic role of HCP’ in network support. Finally, while consultation patterns and the issue of AMR are treated separately in the literature, the findings here indicate that in lay perspectives these are connected and this is covered in the third section ‘a mixed understanding of AMR’. (Table 2).

**Table 2:** Network typology and key themes

Name	Network type	Rehearsed self-care strategies	Mixed understanding of AMR				Temporary / Strategic Role of HCP		
			AMR is where resistance occurs in the …		Concern				
		Support for self-care comes from…	Bacteria	Human body	Population	Individual	% HCPs network member	Access to GP…	Help seeking trigger
Bev	Diverse	Family	X		X	X	25^a^	Delay	Severity
Florence	Diverse	Family (HCP)			X	X	20^a^	Delay	Holiday
Emilia	Diverse	Friends			X	X	24^a^	Delay	Severity
Dianne	Diverse	Friends and Family (HCP)	X		X		13^a^	Delay	Persistence
Ruth	Diverse	Spouse / friends (with children)	X	X			13	Delay	Severity
Simon	Diverse				X	X	13	Immediate	LTC treatment plan
Pearl	Diverse	Family / Friends	X	X	X		26	Delay	Severity
Rachel	Diverse	Friends (with children)			X	X	24^a^	Minor delay	Holiday
Rose	Family and friend centred	Family (HCP)	X		X		16^a^	Minor delay	Reoccurring illness
Gail	Family and friend centred	Friends (HCP)	X		X		75^a^	Delay	Persistence
Lynn	Family and friend centred	Homeopath			X	X	36	Delay	Severity
Emma	Family and friend centred	Spouse and Friends (with children)			X	X	5	Minor delay	Severity
Victoria	Family and friend centred	Spouse	X	X	X	X	19	Immediate	Holiday
John	Family and friend centred	GP	X		X		35	Immediate	LTC treatment plan
Betty	Family and friend centred	Family and GP			X	X	29	Immediate	Severity
Anna	Family and friend centred	Family and Pharmacist	X		X		18	Delay	Persistence
Charlotte	Family and friend centred	Family			X	X	14	Minor Delay	Severity
Sue	Family and friend centred	Spouse			X	X	36	Minor delay	Severity
Caroline	Family and friend centred	Spouse	X		X		37.5^a^	Delay	Severity
Sophie	Family and friend centred	HCP and Family			X	X	43^a^	Delay	Severity
Hugh	Restricted	GP	X	X	X		37	Immediate	LTC treatment plan
Laura	Restricted	HCPs and Spouse			X	X	25	Minor delay	Severity
Pete	Restricted	Spouse	X		X		40	Delay	Severity
Ronald	Restricted	HCP			X	X	100^a^	Immediate	Severity

^a^family or friend are also health care professionals (HCP)

### Rehearsed self-care strategies

Individuals draw upon familiar and well-rehearsed self-care strategies, and those strategies advised by their network members, to manage what they consider to be the more immediate risk to their current health that would occur if action around amelioration is not taken.

*'I thought I probably had a UTI, because of the symptoms. I tried to do the usual thing of flushing through for a couple of days’ (Anna).*

Personal networks can offer an individual support in the management of health risks that can delay the individual in consulting the GP or using other healthcare resources.

*‘Once I’ve exhausted my immediate [network members] then I’ll go to the GP, or I’d go to NHS choices if I couldn’t talk to my friends’ (Gail).*

Specifically, diverse personal networks can be advantageous as they offer increased capacity for the individual to harness a range of knowledge and resources in support of self-care practices. Similarly, in the case of both diverse and family and friend centred networks network members provide valued support by engaging in ‘*information exchange’ (Anna)* and *‘by providing advice on previous conditions’ (Charlotte)*. Network members are therefore an important source of information that can support an individual in the pre-consultation stage, especially where network members may have experienced similar.

*‘My cousin [name] because he’s been going through the same kind of thing as I have, so we talk a lot about stuff’ (John).*

Special value was placed on information provided by network members who had shared experiences as this sharing added credibility to the advice they gave. The more diverse a personal network the more potential there is for the individual to encounter experiential expertise, which acted as a powerful source of reassurance contributing towards a delay in consultation. This was especially the case for parents who were more likely visit the GP sooner for their child’s health than their own, but would delay any visit if they were able to draw upon shared experiences of network members.

*‘I’ll put my school mum friends here because you chat to them “has your child had this” and regular friends with children so you go “oh has your child had this, that or the other*” *(Lyn).*

The support a diverse network offers an individual is relevant to the capacity of that individual to manage prior to seeing a HCP. In contrast where a person’s network is more restricted, and they are more isolated, the help of statutory health services may be sought earlier. Whereas diverse networks expose the individual to a range of self-care strategies and advice, having a more restricted networks does not afford the individual this opportunity.

*‘It’s going to have to come down to the GP because I keep myself to myself’ (Ronald).*

It follows then that in the absence of a range of network members to offer reassurance or advice, individuals with restricted networks seek professional help sooner than someone with a more diverse network.

Furthermore, the mere presence of a spouse offers valued support and is key in supporting the individual’s capacity to self-care that contributes towards a delay in using healthcare services. Nineteen of the 24-people had a spouse in their personal network. Described as ‘*the first person [they’d] go to if [they] had a problem’ (Joan)*, spouses were able to advise about ‘*going to see the GP and getting checked out’ (Florence)*. Spouses, across all network types monitor the effectiveness of self-care strategies and they are also influential in the triggering of consultation. Where diverse networks lead to a delay, spouses are integral to supporting individuals and contribute towards an element of delay.

### The temporary and strategic role of the HCP in network support

Across all network types the GP was listed as an important network member. All but one person listed the GP on their personal network maps. The expertise of GPs is valued by individuals with all network types because ‘*they’re not someone who’s done a three-week course, it’s someone who’s obviously gone to university’ (Emma)*. People value the intervention of the GPs because ‘*they know what they’re doing’ (Simon)*, and so the HCPs role in a network is strategically sought by individuals because ‘*they’ve [GPs] got the medical background’ (Bev)*.

Individuals with informal access to HCPs in their personal network are in an advantageous position as they draw upon this expertise prior to formally consulting a HCP (typically the GP).

*‘Because they’ve got the medical background I would certainly ask you know these people [friends who are nurses] but I would also make a phone call to these two [friends who are nurses]’ (Bev)*

Having access to HCPs in lay environments was most closely associated with having a more diverse networks. Whilst individuals did not seek diagnosis from these network members (informal HCP ties), the reassurance their medical advice provided may be a contributing factor towards a delay in formal health seeking.

For individuals with both diverse and family and friends centred networks help seeking from HCPs was strategically sought after a period of delay (self-care). The role of the HCP (usually the GP) at this stage was temporary, particularly so for people with diverse networks who purposively visited HCPs to seek clarity over their illness experience.

*‘One of the reasons you go to see the GP is that you want them to say, “you’ve definitely go this, you definitely need that, because I’m a professional”’ (Camellia)*

At the point where risk is greater than the capacity of the individual, and their network to manage, HCPs become active network members. In the case of people with diverse and family and friend networks the illness experience persists leading to them question the severity of the illness and therefore seek assurances from the GP.

*‘Once it’s unmanageable then that’s when you go to the doctors…it’s down to the GP to help’ (Charlotte)*

People with more diverse networks who are more likely to have delayed before help seeking may be more likely to use the GP strategically to obtain antibiotics because they have gone through a period of self-care.

*‘I try the usual thing for a couple of days and then I go to the GP for antibiotics if nothing has worked’ (Pearl).*

While GPs remain gatekeepers to antibiotics, and in some instances, are *‘a means to an end if [individuals have] gone through everything else’ (Gail)*, it is also important to individuals with diverse networks that assurances are given that the antibiotics prescribed are the correct response.

*‘They went “we don’t know what it is. Here’s some antibiotics, it may be a UTI”, I didn’t have much confidence so I waited’ (Caroline).*

Individuals with diverse networks are more likely to question HCPs if the role the individual requires them to fulfill is unsatisfactory, that is, if the HCP is unable to provide sufficient clarity. Whereas those individuals with restricted networks demonstrate more dependency and reliance on HCPs, as well as prioritising HCPs expertise over others.

*‘In health matters they’d be behind the GP. GPs the person I would listen to most’ (Hugh).*

Individuals with more restricted networks are less likely to delay in help seeking from formal HCP network members and the expertise of HCP fulfills an informative role that non-HCP members in diverse networks offer the individual. Individuals with restricted networks are also less likely to question HCPs who fulfill more of a central role in their network.

*“It would have been sort of somewhat bizarre to have gone down there and seen the doctor and then reject the advice” (Pete).*

### Understanding of AMR

As a concept, as a public health message, all respondents regardless of their network type considered AMR a societal issue that required action. Thus, this is suggestive that public health messages regarding AMR disseminated via media outlets have some success in reaching the population.

*‘it has been in the media quite a lot…the more you take antibiotics your body doesn’t respond to them’ (Laura).*

Even for individual with more diverse network type media outlets were a key source of raising awareness.

*‘I only know what I’ve heard in the media, particularly news bulletins and articles I’ve read. It’s a national problem’ (Pearl).*

Across all network types there was an awareness that AMR is a public health issue caused by over use/ misuse of antibiotics and this awareness had influence over health seeking behaviour. There was a concern that people *‘didn’t want to be part of the problem’ (Joan)* and they *‘didn’t want to add to the issue’ (Alison)*.

The nature of the exact problem of AMR varied among respondents. Distinctions between two understandings of AMR were common. One understanding was that ‘*the germs are getting used to antibiotics*’ *(Pearl).* Understanding AMR to be about mutation of the microbes that cause an infection aligns more closely with messages that are given by public health bodies.

*‘The microbes mutate to the point where the antibiotics are no longer effective. Originally the antibiotics manage to sort of penetrate the microbes’ defences, but the microbes have evolved to counter that weakness’ (Pete).*

The second understanding was regarding personal immunity where AMR was understood to be when the antibiotics *‘destroy the immune system’ (Dianne)*. This was also seen across all network types.

*‘when your body builds up resistance to that specific antibiotic. My body will build up the resistance to the amoxicillin working in the way it should so that when I take it, it has no effect on actually doing the job it should. I’ve seen it in media outlets, like in the front page of my husband’s BMJ magazine’ (Florence).*

*‘Where your body has built up resistance against them [antibiotics] so they don’t take effect anymore’ (Laura).*

It was a case that for respondents, regardless of network type, they developed an understanding of AMR typically via media outlets, rather than networks members. This illuminates how although a societal problem and that messages in the media are being picked up on by the public, AMR may not be subject to contagion as it is not discussed within personal networks.

*‘It’s not my place to tell them…I know what I feel but then my choice isn’t other people’s’ (Bev).*

*I have had the odd acquaintance say to me “I went to the doctors to get some antibiotics”. I don’t voice my opinion because you don’t want to loose your friends…but I do think to myself “well, that’s pretty selfish”’ (Hugh).*

Individuals across all network types talked of refraining from advising network members on antibiotic use. Also, respondents stated that discussions about antibiotic use is the person’s concern or it is for the GP to advise.

## Discussion

The results from this study help to establish the relevance of social network analysis for understanding primary care related phenomena through defining the role and influences of a personal network in the process of help seeking for antibiotics. Although all respondents were aware that AMR posed a public health risk, there were two distinct understandings of AMR that had no clear network influence. Where network influence was most prominent was around help seeking behaviour for antibiotics.

Diverse networks may offer the individual advantages over more restricted networks and this has influence over help seeking behaviour. Most notably a diverse network offers access to knowledge and resources that can support an individual to self-care away from formal HCP input. The individual finds themselves in a more advantageous position than those with more restricted networks for they have increased capacity to manage illness. As such the role of HCP for people with diverse networks is to offer a degree of clarity when an illness persists, and the self-care strategies advised by network members do not appear to ease symptoms. Whereas for individuals with restricted networks the role of the HCP is activated sooner for the reassurance and advice a diverse network would otherwise offer. An individual with restricted network lacks the necessary support and thus seeks help from HCPs, with whom they are more reliant upon, sooner than those with diverse networks.

### Comparison with existing literature

The findings from this study echo what is known about the influence of social networks on how an individual responds to symptoms [35], how they manage illness [36] and on help-seeking [17]. It also adds further support to the existing understanding of how advantageous diverse networks can be for an individual. Specifically, how weaker ties offer access to knowledge and resources [27] that in this instance can support a delay in help seeking for antibiotics. In support of Rogers et al. [37] who found patients engage in a process of self-care before consulting a GP, this study illuminates how those with diverse networks are able to self-care for longer before seeking formal help. What is known about help-seeking behaviour is that the process is circular [12], and fraught with many considerations including illness uncertainty, anxiety around wasting doctors time and service use entitlement [38–40] and influences of family and friends [17, 41]. This study supports Cromme et al’s [42] who found those who seek help earlier have the belief the GP can provide reassurance and symptom alleviation advice. This study expands on this by demonstrating it is individuals with restricted networks who are more likely to seek this level of support at an early stage. Finally, the public continue to understand the threat AMR poses [43], are increasingly aware of the limitations of antibiotics [44] and continue to trust GPs to determine antibiotic use [45]. Furthermore it demonstrates the public continue to understand AMR to be about individual loss of responsiveness [46] brought about via personal over use [47]. Through demonstrating the little influence informal network members have on antibiotic attitudes and use, the important role GPs as gatekeepers occupy regarding antibiotics and knowledge is highlighted.

#### Strengths and limitations

The use of personal mapping exercise has proven insightful in a chronic condition health context, and it appears to have been helpful in this study. The exercise provided a platform for discussion and reflection the method has enabled the role of networks in attitudes and behaviours around antibiotics to be highlighted in a way other studies have not. Some caution should be noted in that the relatively self-selecting nature of the sampling strategy may have favoured people with a personal interest in, or strong view of, AMR to respond to the study invitation. Whilst efforts were made to invite individuals from practices in different socio-economic areas, the sample is skewed towards a relatively affluent, white British representation. It is therefore unclear what the network effect beyond these demographics might be and thus how generalisable the results are. While the self-selecting nature of the recruitment strategy can (and has) led to some bias in the sample it is also a point of reflection for this study. That is, it raises questions around how far AMR is a prominent issue and concern in the minds of the general public. These strengths and limitations inform the recommendation for research.

#### Implications for practice and research

With the UK’s current policy focussed on a dramatic reduction in inappropriate antibiotic prescribing by 2020 this study has three implications for consideration. The findings of this study supports existing literature documenting the effects on health and wellbeing. Therefore, practitioners may wish to consider using knowledge of patients’ personal networks and their pathway to consultation to modify their consultation approach, and to mitigate against some of the uncertainties and perceptions around prescribing behaviour [48]. Firstly, for example practitioners could consider using their resources to focus on advising on self-care strategies and building the support system around the most isolated people who are likely to consult early in the illness course. GPs may wish to facilitate building the support network around an isolated individual by signposting to community pharmacists for example or to link workers and interventions facilitating network engagement who can provide this support [25]. Secondly, the use of delayed prescribing maybe more appropriate in these instances. Thirdly, for individuals with more diverse networks GPs, through understanding the network effects identified, will be aware that these individuals are likely to have had an extended period of waiting and self-care prior to consultation. The strategies here are likely to differ to those mentioned above. A suggestion is that at this juncture those with diverse networks might benefit from more specific approaches for instance the use of near patient tests or targeted prescribing using decision rules [49, 50]. Merely offering additional self-care advice by way of the TARGET leaflet for example, is likely to repeat much of what has already been tried by the patient.

It would be prudent to replicate these findings in further studies, firstly to seek greater diversity in the sample, and to investigate the network effects across different socio-demographics. Secondly should these findings be confirmed it would helpful to devise a short but effective mechanism by which GPs can assess patients’ social network. Thirdly research should test the hypothesis that advice approaches based on network typology are more effective than a generic approach.

## Conclusion

Antimicrobial resistance is a global threat that public health measures have attempted to control via reducing prescribing in primary care; the site of 74% of all prescriptions. However, measures do not take into account the social network influences on attitudes and behaviours. This paper has highlighted the importance of taking a social network approach to the issue. The paper concludes therefore that the type of network holds the potential to affect an individual’s help seeking behaviour for antibiotics, with individual’s with more diverse networks able to delay their help seeking. This information can be used by physicians to help tailor their prescribing approach and to guide when to issue an immediate, delayed or no prescription.

## Additional file

Additional file 1: Interview guide. (DOCX 72 kb)

## Abbreviations

AMR: Antimicrobial resistance
HCP: Healthcare Professional
NIHR CLAHRC: National Institute Health Research Collaboration for Leadership in Applied Health Research and Care
RTI: Respiratory tract infection
TARGET: Treat Antibiotics Responsibility, Guidance, Education, Tools
UTI: Urinary tract infection
