# Supplementary material for: Help seeking for antibiotics; is the influence of a personal social network relevant?
Source: BMC Fam Pract. 2019 May 14;20:63. doi: 10.1186/s12875-019-0955-2 (PMC6518744; doi:10.1186/s12875-019-0955-2)
Supplement: Supplementary file 1 — Interview guide. (DOCX 72 kb) [file 12875_2019_955_MOESM1_ESM.docx]

**Title:** ‘Back-up’ or ‘just in-case’ prescription for antibiotics: what does it mean to you?

**Introduction**

Researcher to introduce self, check the participant has read the information sheet given sent to them. Gain written consent. Ask if audio-recording can be turned on remind them when it is that the interview is being recorded.

- Thank individual for taking part.
- Concerned with understanding how the decision regarding antibiotic use was made, and the influences.
- No right or wrong answers.
- Remind of research rights- right to withdraw, pause or not answer a certain question if don’t want to.

**Personal experience**

Can you think about the last time you had to make a decision whether or not to take antibiotics, I want you to think about when you started to feel unwell.

- Can you tell me when this was (approximately)?
- Can you tell me how you were feeling at this time?
  - - What were your symptoms?
    - How long did this go on for?
- How did you respond to this, how did you manage the symptoms you were experiencing / how did you manage your health?
- What made you see your GP?
  - - Did you talk to anyone about going to see the GP?
    - And when was this?

**Antibiotic use**

- What did the GP / nurse tell you when you were prescribed antibiotics, can you remember?
  - - Did they tell you to wait to see if your symptoms got better before taking the antibiotic?
- Did you use the antibiotics prescribed to you?
  - If yes, how long after the prescription was issued did you use them?

**General knowledge of antibiotic and AB resistance**

I wonder if we can turn to antibiotics more generally.

- Can you tell me what you know about antibiotic resistance?
  - - What do you understand by antibiotic resistance?
    - What does antibiotic resistance mean to you?
- Can you tell me how you have come to know this?
  - - Who / what has helped you to think this?

**AMR as the problem**

In bringing this to a close then could I ask you a few more general questions, and there are no right or wrong answers to this.

- What do you think has contributed towards AMR/AR?
- Do you have a view of what has caused AMR/ AR?
- What do you think should happen to stop the increase in AMR/ AR?
- What needs to change to combat this issue?
  - What do you think GPs could do?
  - What could patients do?
    - What would patients need to help them do/achieve this?
  - Is there anyone else who might be able to help combat this?
    - Does the Government have a role to play? The Media? Pharmacists? Etc.

**Closing out**

Thank you for talking to me:

- Do you have anything else to add?
- To you have any questions for me?
- Thank you.
